# Supplementary material for: Adopting sustainable innovations for remote access to TB and HIV care in South Africa
Source: PLOS Glob Public Health. 2024 Oct 16;4(10):e0003792. doi: 10.1371/journal.pgph.0003792 (PMC11482720; doi:10.1371/journal.pgph.0003792)
Supplement: S1 Table — (DOCX) [file pgph.0003792.s001.docx]

S1 Table. Summary of data fields extracted and grouped by main intervention theme (March 2020 and December 2021.

| **Theme** | **Country** | **Sample size** | **Setting** | **Outcome or goal measure** |
| --- | --- | --- | --- | --- |
| Patient support | Myanmar | - | 12 townships | More than 95% of DR-TB patients have received monetary support in this manner. These interventions have reduced loss to follow up, hardship and suffering of DR-TB patients, including those co-infected with COVID-19 or identified as contacts. |
|  | South Africa | 232 | - | A total of 232 telephone consultations were held. Participants (patients with rifampicin-resistant TB) reported a high degree of satisfaction with the programme and reported that they felt “less isolated” and better “cared for”. |
| Virtual models | Brazil | 185 patients | primary health-care units | Two or more follow-up calls have been made to 41.6% of those enrolled (TB). Of these, 96% answered at least 50% of the programmed calls. |
|  | Brazil |  | Four primary health-care units and at the Hospital | The app reduced the need for DOT home visits by 43%, the number of directly supervised medication intakes increased dramatically with the use of VOT, with 80% of all planned intakes validated. |
| Treatment | Dominican Republic | 1381 patients | - | Monitoring was conducted in two phases: 79% of patients were contacted in May 2020 and 68% in August 2020. 1381 TB patients, of whom 1095 were contacted (79%). Of these patients, 940 were still in treatment at the time of the call (86%). Of the 940 patients treated, 749 (80%) reported taking medications under home DOT, 20% reported taking medications in health facilities, and 433/940 (46%) reported no verified DOT. |
|  | South Africa | - | Metro district | Children with long-term health conditions (LTHCs) such as HIV and TB were given a one- to two-month supply of medication and ongoing pre-packaged supplies were delivered to their homes by community health workers (CHWs). |
|  | South Africa | - | Farms | Hlokomela provided four types of outreach activities: provision of personal protective equipment; workplace and community screening services; training and awareness activities; and distribution of food. Hlokomela was able to sustain PHC service provision, but also maintained basic occupational Health services and by July 2021, more employers recognised the value of Hlokomela’s agency for health service provision. |
|  | Uganda | - | - | *The project is under way*. So far, it has been learnt that adapted, patient-centred digital adherence technology has resulted in fewer clinic visits by remote monitoring of treatment adherence (for TB), presenting an important strategy for reducing clinic congestion during the pandemic. |
|  | Kenya, Malawi and Zimbabwe | - | Purposively selected  18 health facilities in Nairobi, 8 in Lilongwe and 10 in Harare. | The overall numbers of people presenting with presumptive pulmonary TB decreased, ranging from 31% decrease in Kenya to 46% decrease in Malawi. Registered for TB in each country showed parallel decreases. TB treatment success was less severely affected with Kenya seeing a slight improvement in the COVID-19 period compared with the pre-COVID-19 period. |
|  | Guatemala | - | - | 756 patients received institutional DOT, 362 home-based DOT, 65 community-supported DOT, 210 family-supported DOT and 20 VOT. Of 169 people with TB screened for COVID-19, 52 were co-infected with SARS-CoV-2. |
|  | Pakistan | - | - | 98% of TB patients were able to continue their treatment without interruption in districts covered by the programme. Between 1 April and 23 December 2020, 29,445 patients were diagnosed and treated, 9,785 patients received various types of follow-up interventions, and 41,389 household contacts were screened for TB. |
|  | Philippines | 186 patients | - | Enrolment for the standard short all-oral regimen increased to 364 patients between April and June 2020 and further increased to 467 patients between July and September and to 449 between October and December, for a total of 1466 patients initiated on this treatment in 2020. |
|  | Uruguay | 568 | Health centres | 568 patients were treated. In the first survey, 227 of 251 patients (90%) were monitored, of whom 118 (52%) by VOT. During the second survey, 263 of 317 patients (83%) were monitored, of whom 145 (55%) by VOT. In Montevideo, where 55% of the country’s TB cases are treated, 98% of cases were monitored, with 58% by VOT. The suitability of this intervention was considered positive by 70% of health workers and 60% of patients; 18% of the health workers did not support the intervention, and 12% were neutral. |
|  | Uganda | 3075 | 17 districts of Uganda | 3075 adults with pulmonary TB who initiated first-line treatment at the study sites, 2618 (85%) were enrolled on 99DOTS. Altogether, 96% of the expected daily doses of anti-TB medications were confirmed as taken (70% via patient phone calls to the 99DOTS platform and 26% via follow-up phone calls or home visits by community health workers when patients did not make calls). Community health workers made 6747 phones calls to 1466 (56%) patients (median, three per patient) and 1394 home visits to 680 patients (median, one per patient). 99DOTS was well received by both patients and providers. |
| Screening, testing and diagnosis | Ethiopia | 1629 patients | - | Of those screened, 326 (20%) had some form of TB and were directed to treatment. |
|  | India | 2500 patients | - | The project has demonstrated some early successes: (i) reducing the turnaround time for laboratory results by changing from hard-copy to SMS delivery; (ii) recording patients’ longitudinal treatment journey; and (iii) introducing refill reminders to reduce disruptions in treatment. One of the reasons for not accepting this service was reported as stigmatization, as patients did not want to reveal their illness to other. |
|  | India | - | The target population are the poor and vulnerable. | 1817 cases of TB were diagnosed in our intervention area, representing 76% of all cases in the district. The overall rate of treatment initiation was > 95%, and the treatment success rate was 90%. |
|  | India | Enrolled and trained 755 providers in TB care | - | From a target population of 893 086, informal providers referred 9245 patients, of whom 8939 (97%) presented for testing and 1147 were diagnosed with TB. |
|  | India | - | - | Results will be analysed in the third quarter of 2021. |
|  | Philippines | - | Three COVID-19 facilities | Preliminary data from 25 August–30 September 2021 showed a total of 1510 people screened for TB (1106 in swabbing areas and 404 in isolation wards) among 5818 people served in the three COVID-19 health facilities. The final analysis of results is scheduled in 6 months. |
|  | Mozambique | - | TB patients and the general population, children under 5. | 2174 patients were telephoned to strengthen their adherence and to trace contacts; 11 patients who had been lost to follow-up were reconnected to services; however, 234 patients could not be supported with telemedicine because of difficulty in accessing telephone services. The number of children under the age of 5 who started TB preventive treatment had decreased by 46.5% |
|  | Philippines | - | - | 21 304 vaccinees (for COVID-19 vaccination) were screened for TB by symptoms and chest radiography, of whom 1365 (6%) were presumed to have TB. A total of 915 (67%) vaccinees with presumptive TB were tested with Xpert MTB/RIF®, of whom 259 were diagnosed with TB and enrolled on treatment; the remaining 450 people refused consent to give a sputum sample |
|  | Philippines | - | All patients presenting for SARS-CoV-2 | 2495 individuals were tested for SARS-CoV-2 and TB. Of these, 401 were presumed to have TB. All were tested with GeneXpert, of whom 88 (4%) tested positive for TB. TB case finding during COVID-19 screening yielded a higher rate of case-finding (1 person diagnosed for every 28 people screened bacteriologically or clinically) than active TB case finding |
|  | Nigeria | 1931 people enrolled | - | 1928 (99.8%) screened with CAD4 TB X-ray, 83 were found to have presumptive TB, and 11 (13%) cases were diagnosed with GeneXpert. From the people screened, 1252 nasopharyngeal swabs were collected and tested for SARS COV-2, and all were screened for HIV; 183 (15%) cases of COVID-19 and 12 (1.0%) cases of HIV infection were diagnosed. |
|  | Russian Federation | - | The project is focused on vulnerable groups: high-risk contacts of TB patients, homeless people and people living with HIV. | 3500 people in the target groups had been screened for TB. Of 420 people suspected of having TB disease, 21 (5%) had confirmed active TB. Of 398 people eligible for TB preventive therapy, 280 (70%) completed a full course, 40 (10%) stopped because of adverse events, 51 (13%) discontinued therapy, and 27 (7%) are still under therapy. One person who declined and one person who discontinued TB preventive therapy but none of those who completed or continued treatment developed TB. The study showed that homeless people were at increased risk of TB: 71% of people diagnosed with active TB and 33% of those lost to follow-up were homeless |
| Health-care worker support | Colombia | More than 1000 health workers | Virtual training | More than 1000 health workers were trained virtually |
|  | Paraguay | Approximately 500 health professionals | - | The impact of this intervention is still being evaluated, however, a 20% increase in monthly TB case-finding has been observed. |
|  | Ukraine | 150 | 10-day virtual training | 150 TB health-care staff received training (in prevention of emotional burn-out and self-help technique). Direct feedback from the trainees showed improved emotional competence and psycho-emotional state; greater awareness of the symptoms of professional burn-out and self-help techniques; greater awareness among the leaders of health institutions of the importance of 24 programmes for the prevention of burn-out; and greater awareness of mental health among health-care staff of TB institutions in Ukraine. |
|  | Uruguay | 19 doctors, 139 nurses and 8 administrators | Medical, technical and administrative health workers | Four virtual training sessions on TB and COVID-19 on Zoom, focusing on prevention measures and strategies to maintain NTP operations, in which 86% of the targeted audiences participated. |
| Monitoring and evaluation / reporting | Zambia | 80 | - | All the districts implemented best practices in case finding. Notifications of TB increased consistently thereafter, resulting in notification of more cases (7.5–10.8% increase) in 2020 than in 2019, 2018 and 2017 |
