# Supplementary material for: Adopting sustainable innovations for remote access to TB and HIV care in South Africa
Source: PLOS Glob Public Health. 2024 Oct 16;4(10):e0003792. doi: 10.1371/journal.pgph.0003792 (PMC11482720; doi:10.1371/journal.pgph.0003792)
Supplement: S2 Table — (DOCX) [file pgph.0003792.s002.docx]

S2 Table. E-survey

| 1 | Unique study number | | Assigned by REDCap | |
| --- | --- | --- | --- | --- |
| 2 | Salutation | |  | |
| 3 | Name | |  | |
| 4 | Surname | |  | |
| 5 | Email | |  | |
| 6a | Contact number | |  | |
| 6b | Can we contact you at if we need more information? | | - Yes, you may contact me - No, please do not contact me | |
| 7 | Sector | | - Private - Public - Government - NGO - Community based - Civil society | |
| 8 | Name of organization / facility / department | |  | |
| 9 | Designation / Role | |  | |
|  |  | |  | |
| 10 | Did your organization / facility / department implement any changes / adaptations / innovations to HIV or TB service delivery in response the COVID-19 pandemic? | | - Yes (Skip to Q13) - No (Skip to Q28) - Don’t know | |
| 11 | Is there someone else in your organization / facility / department that would know if changes / adaptations / innovations to HIV or TB service delivery were implemented in response the COVID-19 pandemic? | | - Yes - No (Skip to Q28) - Don’t know (Skip to Q28) | |
| 12 | If yes to Q11, can you provide the name and contact email for this person? | | Name __________________  Email __________________ (Skip to Q28) | |
| 13 | If yes to Q10, can you select a key theme(s) that best describes the change(s) implemented? | | | |
| 14 | - Screening, testing and diagnosis | - How treatment is provided (including preventative therapy) | - Community model | - Virtual model |
|  | - Medication collection and delivery | - Patient support | - Awareness, education and patient empowerment | - Other |
|  | - Clinic visits | - Health-care worker support | - Monitoring and evaluation / reporting | - Don’t know |
| 15 | If other or don’t know, please specify | | | |
| 16 | If any key theme was selected, please explain the changes / adaptations / innovations to HIV or TB service delivery in your own words.      2.  3.  4.  5. | | | |
| 17 | Where was this change / adaptation / innovation implemented (province, district, sub-district, facility)? | | *Enter in the order in which these were listed in Q16.* | |
| 18 | How many clients received the change / adaptation / innovation? | | *Enter in the order in which these were listed in Q16.* | |
| 19 | What patient population was included in the change / adaptation / innovation? (HIV, TB, MSM, stable PLHIV, key population etc.) | | *Enter in the order in which these were listed in Q16.* | |
| 20 | When was the start date of the change / adaptation / innovation? | | *Enter in the order in which these were listed in Q16.* | |
| 21 | When was the stop date of the change / adaptation / innovation? | | *Enter in the order in which these were listed in Q16.* | |
| 22 | Has your organization/facility/department continued to implement this change / adaptation / innovation as part of the standard of care? | | *Enter in the order in which these were listed in Q16.*   - Yes - No - Don’t know | |
| 23 | Did your organization / facility / department receive funding for this change / adaptation / innovation? | | *Enter in the order in which these were listed in Q16.*   - Yes - No (Skip to Q25) - Don’t know (Skip to Q25) | |
| 24 | If so, where was it funded from? | | *Enter in the order in which these were listed in Q16.* | |
| 25 | Does your organization / facility / department have any data (routine or otherwise) to support the feasibility, acceptability or efficacy of the change / adaptation / innovation to HIV or TB service delivery during the COVID-19 pandemic? | | *Enter in the order in which these were listed in Q16.*   - Yes - No (Skip to Q28) - Don’t know (Skip to Q28) | |
| 26 | If yes to Q25, please indicate what data is available | | *Enter in the order in which these were listed in Q16.*   - Electronic - Paper - Both paper and electronic - Don’t know | |
| 27 | If yes to Q26, please describe if this data is available and if you can share it with others outside your organization / facility / department? | | *Enter in the order in which these were listed in Q16.*   - No - Yes, no restrictions - Yes, restrictions apply - Don’t know | |
| 28 | Did your organization / facility / department implement any **other programmatic** changes / adaptations / innovations to service delivery during the COVID-19 pandemic?  *[Examples include integrating family planning or non-communicable disease management]* | | - Yes - No (Skip to Q30) - Don’t know (Skip to Q30) | |
| 29 | If yes, please describe the changes / adaptations / innovations | |  | |
| 30 | Do you know of **any other organizations** / facilities / departments that implemented any changes / adaptations / innovations to HIV or TB service delivery in response the COVID-19 pandemic? | | - Yes - No (Skip to Q32) - Don’t know (Skip to Q32) | |
| 31 | If yes, please provide the name of the organization / facility / department and briefly mention the change / adaptation / innovation. | |  | |
| 32 | Would you be willing to participate in an in-depth interview to explore some of this in more detail? | | - Yes - No | |
