# Supplementary material for: Adopting sustainable innovations for remote access to TB and HIV care in South Africa
Source: PLOS Glob Public Health. 2024 Oct 16;4(10):e0003792. doi: 10.1371/journal.pgph.0003792 (PMC11482720; doi:10.1371/journal.pgph.0003792)
Supplement: Attachment — Submitted filename: Review Comments to the Author .docx [file pgph.0003792.s004.docx]

Review Comments to the Author

Reviewer #1: A very good paper and quite informative. It would be very helpful if the same study could be reproduced if other databases other than MEDLINE were to be used. Furthermore, feasibility studies on the effectiveness, sustainability and cost effectiveness would help guide on how best the ICTS can be best integrated in existing programs particularly TB and HIV programs.

Thank you for these very helpful comments. We agree that it would be helpful if the same study could be used with other databases than MEDLINE and will look at this possibility in the future. We chose MEDLINE as it is the primary component of PubMed and contains more than 31 million references to journal articles (<https://www.nlm.nih.gov/medline/medline_overview.html>). Furthermore, we also followed the guidance from Smela et al., 2023 (https://doi.org/10.1080/20016689.2023.2241234) and Dobbins et al., 2017 (https://www.nccmt.ca/capacity-development/rapid-review-guidebook [<https://www.nccmt.ca/capacity-development/rapid-review-guidebook>) which state that solutions for rapid literature reviews include narrowing the scope or searching a limited number of databases and other sources.

We acknowledge this as a study limitation (Line 487-490; “we only searched one database (MEDLINE) for the rapid review, but since this was to inform the key intervention groups/themes we considered this to be sufficient, particularly once we achieved saturation (i.e., no new themes were found).”

Regarding the point about **feasibility, effectiveness, sustainability and cost-effectiveness**. We agree that these are very important indicators that should be considered. However, at the time of the study we found a lack of data on these indicators (Line 228, 497 and 499), so we were unable to make recommendations about specific interventions. Although there is growing evidence, more data is needed on the clinical efficacy, cost effectiveness and feasibility of interventions.

Reviewer #2: An interesting study but it must be -organized a bit further with more rigorous data documentation including actual data disaggregation with charts and tables of the literature review and interventions. Making the distinction between programmatic and differentiated service delivery interventions and providing greater insight on the human resource capacity challenges would be helpful.

We greatly appreciate these comments and agree that additional charts and tables of the literature review and interventions would be helpful. We have decided to include **Supplementary Table 2** summarizing some of the data fields that were extracted from the rapid review. We hope this provides greater insight based on your suggestions.

Reviewer #3: Coming out of the COVID-19 pandemic, it is crucial to publish high-quality review articles to stimulate progress in advancing major initiatives on dual TB and HIV epidemics. The article's premise offers an opportunity.

This is a well-written manuscript that purports to focus on the importance of implementing several initiatives to adapt the service delivery model in HIV and TB in response to the COVID-19 pandemic. A detailed analysis of these initiatives globally focused on the S. African experience is presented, followed by an exciting discussion about practical implications for the country to maintain and extend best practices for integrating HIV and TB services to other services, medication delivery, and telemedicine after the COVID 19 pandemic, highlighting the importance of additional cost-effectiveness data for sustainability of selected interventions.

I have a few comments as follows:
1. Abstract; Rows 69 - 70: Suggest elaborating on the meaning of “services can be delivered outside of health facilities”. I also believe that the paragraph between rows 69 to 75 needs to be more aligned with the key points presented in the discussion section, such as the maintenance of programs post-pandemic.

Thank you for these suggestions. We have taken your advice and clarified the meaning of rows 69-70 in the abstract. We have also made rows 69-75 more aligned as you suggested as well. We hope our changes are satisfactory.

2. Row 244: Suggest elaborating on the meaning of the "changes" that have been made in response to challenges.

Thank you for this suggestion. We have made these changes as your requested.

3. Row 513: Linked with the comment on the abstract above, I suggest providing more details about the types of services that can be offered outside of health facilities.

We greatly appreciate this comment and have made these changes.
