# Supplementary material for: Adopting sustainable innovations for remote access to TB and HIV care in South Africa
Source: PLOS Glob Public Health. 2024 Oct 16;4(10):e0003792. doi: 10.1371/journal.pgph.0003792 (PMC11482720; doi:10.1371/journal.pgph.0003792)
Supplement: Attachment — Submitted filename: 2nd Round Review Comments .docx [file pgph.0003792.s005.docx]

**2^nd^ Round Review Comments – Response to Reviewers**

Journal Requirements

The reference list has been reviewed and corrected

Reviewer #2: Abstract:
• Perhaps emphasize what can be learned from TB focused DSD services from this study.

Examples of DSD services for TB have been included in Line 69-74

• Could there have been a bias for English language studies that were only reviewed in the PRISMA checklists given the different non-English countries on the WHO high TB Burden list? E.g Russia, Mozambique.

We appreciate this comment and have added it as a limitation (Lines 505-509). However, we believe any bias was minimal, as most scientific literature is published in English. Additionally, since this was a rapid review rather than a systematic one, we took a pragmatic approach by extracting data exclusively from English-language papers.

Any potential bias introduced is believed to be minimal since the literature review was conducted to inform the e-survey.

Results/South Africa
• In terms of implementation the total for district, provincial, and facility levels do not add up to 100% (5/57 Missing) could the rest be unknown

Thank you for noticing this. This has been corrected. 3 were “all” populations and were added to “TB and HIV”. Line 274-275

“Among the interventions reported; 47% (27/57), 11% (6/57), and 28% (16/57) targeted HIV, TB, and HIV/TB integrated service delivery, respectively (8/57 not stated).”

• What were the gaps between the gaps observed in TB/HIV integration in the local setting compared to the global setting in the literature review. This could have been summarized highlighted further in the discussion as seen in Figure 1.

Thank you for highlighting this omission. This point has been added to Line 377-379.

“In South Africa, adaptations primarily focused on enhancing screening, testing, and diagnosis, improving medication collection and delivery, and adopting virtual models. The latter two themes were also among the most commonly implemented worldwide.”

• Cost-effectiveness of the innovative interventions would have been nice to compare the innovation versus existing routine costs for TB/HIV services and is noted in the limitations

Agreed. It was an area we were particularly interested in reporting on, but unfortunately, there is a lack of data.

• Outlining the various successful innovations e.g telemedicine, Medication delivery, multi-month dispensing was very effective.

Thank you for this feedback, we appreciate it.

Conclusion
• One could have stressed more the importance of virtual-based platforms to improve the quality of TB/HIV service delivery, as highlighted in the narrative discussion and results.

Thank you for highlighting this omission. This point has been added to Line 532-534

“Virtual models not only assist in patient management but also empower healthcare workers and facilitate the monitoring and evaluation of TB and HIV programs.”
